# Exploring blood transcriptomic signatures in patients with herpes zoster and postherpetic neuralgia

**DOI:** 10.3389/fcimb.2024.1425393

**Published:** 2024-08-15

**Authors:** Chunliang Wang, Kaiyi Zhang, Yuhan Bao, Ye Liu, You Zhou, Yong-Hua Ji, Hongjie Wang, Zhi-Yong Tan

**Affiliations:** ^1^ Department of Pain Management, Affiliated Hospital of Hebei University/School of Clinical Medicine, Hebei University, Baoding, Hebei, China; ^2^ School of Basic Medicine, Hebei University, Baoding, Hebei, China; ^3^ Department of Anesthesia, Affiliated Hospital of Hebei University/School of Clinical Medicine, Hebei University, Baoding, Hebei, China

**Keywords:** postherpetic neuralgia, herpes zoster, RNA-Seq, blood transcriptome, patient, differential expression gene

## Abstract

Postherpetic neuralgia (PHN) is a common, severe, and hard-to-treat chronic pain condition in clinics. Although PHN is developed from herpes zoster (HZ), the developing mechanism is unknown. A previous study investigated blood metabolomic and proteomic profiling in patients with PHN and HZ. The current study aims to explore the blood transcriptomic signature of PHN compared to HZ patients. Whole blood from eight PHN and 15 HZ patients was used for RNA-Seq analysis. There were 82 and 1,788 genes detected specifically in the PHN and HZ groups, respectively. PHN-specific genes are involved in viral infection, lipid and carbohydrate metabolism, and immune response. For genes coexpressed in PHN and HZ patients, there were 407 differential expression genes (DEGs), including 205 upregulated (UP DEGs) and 202 downregulated (DOWN DEGs) in PHN compared to HZ groups. DEGs are involved in viral infection, type I interferon (IFN), and hemoglobin and oxygen carrier activity. UP DEGs are associated with regulatory T cells (Tregs), activated NK cells, and neutrophils, while DOWN DEGs are associated with Tregs, resting NK cells, and monocytes. The results suggest that the metabolism of lipid, glycan, and nucleotides, type I IFN signaling, and altered neutrophil activation are associated with and might contribute to the development of PHN in HZ. It is also suggested that persistent or altered activation of nonspecific immunity may contribute to the development of PHN from HZ.

## Introduction

Herpes zoster (HZ) develops in about 30% of the world population in their lifetime (without the use of a vaccine), and postherpetic neuralgia (PHN) is the most common complication of HZ (van Oorschot et al., 2021; Curran et al., 2022; Pan et al., 2022). The overall risk of PHN after HZ is about 10%–20%, with the majority of these cases occurring at the age of 50 or older (Delaney et al., 2009; Sampathkumar et al., 2009; Saguil et al., 2017). PHN often refers to neuropathic pain that persists for more than 3 months after the disappearance of the rash and blisters associated with HZ. It is one of the most severe and intractable chronic pain conditions in clinics.

HZ is caused by the reactivation of the Varicella-Zoster virus (VZV). The first VZV infection results in chickenpox, which often self-resolves in 7–10 days. Afterward, VZV becomes latent in dorsal root ganglion (DRG) and trigeminal ganglion (TG) neurons. An increase in age or immunosuppression can trigger the reactivation of VZV, leading to HZ. However, it is unknown how HZ develops into PHN.

Due to the host selectivity (to humans) of VZV, there is a lack of valid animal models for HZ and PHN studies. Previous patient studies involve peripheral blood mononuclear cells, serum molecules associated with inflammation, and postmortem examination of immune cell infiltration in DRG and TG (Gowrishankar et al., 2010; Xing et al., 2013; Mercan et al., 2021; Peng et al., 2022). Particularly, a recent study has investigated blood metabolomic and proteomic profiling in patients with PHN and HZ (Zhou et al., 2022). However, blood transcriptomes have not been studied in these patients. The current study aims to explore the differential blood transcriptomic signatures between PHN and HZ, focusing on the indication for the positive association and development of PHN from HZ.

## Experimental procedures

### Study population

The subjects of the current study were inpatients from October 2022 to May 2023 in the Department of Pain Management at Hebei University Affiliated Hospital. Fifteen HZ and eight PHN patients were included (**Table 1**). Patients’ information was collected, including gender, age, disease course, location of zoster, Visual Analog Scale (VAS), and medical conditions. A regular blood examination was conducted. The inclusion criteria of HZ and PHN are as follows: (1) meet the diagnosis criteria in the “Chinese consensus on the diagnosis and management of herpes zoster (2018)”; (2) have a VAS > 3 at admission; (3) be > 18 years old; and (4) be able to evaluate self-pain and give a VAS score. Exclusion criteria include the following: conditions (such as mental and nervous diseases) that do not allow cooperative treatment. The study was approved by the ethics committee at Hebei University Affiliated Hospital. Written informed consent was obtained from all participants before the study.

**Table 1:** Demographic and disease information of PHN and HZ patients.

	PHN (*n* = 8)	HZ (*n* = 15)	*p*-value
**Average age**	63.875	66.13	0.6788
**Average value of VAS**	7.625	7.93	0.6269
Sex (*n*%)			
Male	*n* = 4, 50%	*n* = 6, 40%	0.6850
Female	*n* = 4, 50%	*n* = 9, 60%	
Location (*n*%)			
Face	*n* = 2, 25%	*n* = 1, 6.7%	0.2688
Neck and upper limbs (cervical nerve region)	*n* = 2, 25%	*n* = 4, 26.7%	> 0.9999
Trunk (thoracic nerve region)	*n* = 4, 50%	*n* = 10, 66.7%	0.6570
**Course of disease**	90–365 days	10–60 days	
Quartile (25%)	90 days	20 days	
Quartile (50%)	105 days	23 days	
Quartile (75%)	232.5 days	40 days	
Chronic disease (*n*%)			
Hypertension	*n* = 4, 50%	*n* = 6, 66.7%	0.6850
Diabetes	*n* = 1, 12.5%	*n* = 1, 6.7%	> 0.9999
Coronary heart disease	*n* = 1, 12.5%	*n* = 2, 13.3%	> 0.9999
Valvular heart disease	*n* = 1, 12.5%	*n* = 0, 0%	0.4211
Lung disease	*n* = 2, 25%	*n* = 1, 6.7%	0.5459
Leukemia	*n* = 1, 12.5%	*n* = 1, 6.7%	> 0.9999
Cerebral infarction	*n* = 1, 12.5%	*n* = 3, 20%	0.6027
Mild depression	*n* = 0, 0%	*n* = 1, 6.7%	> 0.9999
Asthma	*n* = 0, 0%	*n* = 1, 6.7%	> 0.9999

### Transcriptome analysis

Blood samples of 1 ml were drawn using EDTA anticoagulation tubes. After adding 3 ml of TRIzol, mixing gently by blowing, and incubating for 5-min, the mixture was frozen and stored in a − 80°C freezer.

RNA purification, reverse transcription, cDNA library construction, and sequencing were conducted by Shanghai Majorbio Bio-Pharm Biotechnology Co. Ltd. (Shanghai, China) according to the relative instructions of the manufacturer (Illumina, San Diego, CA, USA). RNAs were extracted by TRIzol®. RNA quality was detected by a bioanalyzer (Agilent 5300). RNA quantification was determined by a NanoDrop (ND-2000, NanoDrop Technologies, Carlsbad, CA, United States). High-quality RNA samples (OD260/280 = 1.8 ~ 2.2, OD260/230 ≥ 2.0, RIN ≥ 6.5, 28S:18S ≥ 1.0, > 1 μg) were used for construction of sequencing library.

The transcriptome library of PHN and HZ patients was constructed by Illumina® Stranded mRNA Prep and Ligation from Illumina (San Diego, CA, USA) using 1 μg of total RNA. Briefly, mRNAs were isolated by oligo (dT) beads, and a fragment buffer was used for fragment partitioning. Double-stranded cDNAs were synthesized by SuperScript double-stranded cDNA synthesis Kit (Invitrogen, CA, Wilmington, DE, United States) and random hexamer primers (Illumina, San Diego, CA, USA). DNA end repair, phosphorylation, and “A” base addition were conducted according to the Illumina library construction protocols. cDNA target fragments (300 bp) were chosen at 2% low-range ultra agarose. cDNA amplification was conducted by 15 cycles of PCR using Phusion DNA polymerase (NEB). After quantification by Qubit 4.0, the NovaSeq Xplus sequencer (2 bp × 150 bp read length) was used for sequencing of the paired-end RNA-Seq library.

Pair-end reads were trimmed and controlled for quality using fastp (Chen et al., 2018) with default parameters. Clean reads were compared with the reference genome using HISAT2 (Kim et al., 2015) in the orientation mode. The mapped reads were assembled by StringTie (Pertea et al., 2015) in a reference-based approach for each sample. The expression level of each transcript was calculated by transcript per million (TPM). RSEM (Li and Dewey, 2011) was used to quantify transcript abundances in order to recognize DEGs between two samples. Differential expression analysis was performed by DESeq2 or DEGseq (Wang et al., 2010; Love et al., 2014). DEGs with |log2FC|≥1 and FDR < 0.05 (DESeq2) or FDR < 0.001 (DEGseq) were considered significantly different transcripts. Goatools and Python scipy were used for GO and KEGG and DO analyses, respectively. All data analysis was conducted on the Majorbio Cloud Platform (https://cloud.majorbio.com/).

### qPCR

Reverse transcription was conducted using HiScript®III RT SuperMix for the qPCR kit (Vazyme, Nanjing, China). DNA was first degraded by mixing RNA and 4×gDNA wiper Mix. 5×HiScript qRT SuperMix was then used for reverse transcription of mRNA to cDNA. ChamQ Universal SYBR qPCR Master Mix Kit (Vazyme, Nanjing, China) was used for cDNA amplification. Real-time qPCR was conducted by Lightcycler 96 system (Roche, Basel, Switzerland). Primer sequences are listed in **Table 2**.

**Table 2:** Sequences of primers.

Gene name	Sequences
GAPDH	Forward:5′-CACCCACTCCTCCACCTTTGAC-3′ Reverse:3′-GATGTCGTTGCCTCACCACCTG-5′
IFI27	Forward:5′-ATTGCTACAGTTGTGATTGGAGGAG-3′ Reverse:3′-CGGACATCATCTTGGCTGCTATG-5′
RPL9	Forward:5′-ATGAGACCAGGTGTTGCTTGTTC-3′ Reverse:3′-TTTAACTGTTGTGGCTTGCTGAATC-5′
LCN2	Forward:5′-GCAGCAGAACTTCCAGGACAAC-3′ Reverse:3′-TTTCAGCTCATAGATGGTGGCATAC-5′
SLC25A37	Forward: 5′-CTTCCAGTCCATCCACTTCATCAC-3′ Reverse: 3′-GCCAGCCCGCCTGAGATG-5′
FGFBP2	Forward: 5′-GCCGCAACACAGACCAGAC-3′ Reverse: 3′-GCAGGGCTTGATTCCAGTAAGG-5′
ISG15	Forward: 5′-CCTGCTGGTGGTGGACAAATG-3′ Reverse: 3′-CCGCTCACTTGCTGCTTCAG-5′
LTF	Forward: 5′-CCGCCGTGGACAGGACTG-3′ Reverse: 3′-CGCCAATACACAGAGCACAGAG-5′
BPI	Forward: 5′-CGTCCCTGATGGTGCTGGTC-3′ Reverse: 3′-CCTGCTGGCTGGCGTAGTC-5′
MKKS	Forward: 5′-GATTATACAATAGCCAGGAAGAACTCAAC-3′ Reverse: 3′-GCAGTCAAACAGTCCAAGGTCAG-5′
HMGB3	Forward: 5′-TTACATCACTAAGGCGGCAAAGC-3′ Reverse: 3′-TTCCTCCTCCTCTTCTTCATCTTCC-5′
SIGLEC1	Forward: 5′-AACTTGCTGCGTGTGGAGATTC-3′ Reverse: 3′-TTGAAGGTGGCTGAGGTGGAG-5′
NDUFAF4	Forward: 5′-CGAGCGGAACGGGAAATCAG-3′ Reverse: 3′-CTTTACGAGCAATCTCTCCTTTAACTTC-5′
ESPN	Forward: 5′-CCAACTACGACTCCTGCTCCTC-3′ Reverse: 3′-GTCTGTATGTCTCGATGTACCTGTAC-5′
IFI44L	Forward: 5′-TGGAGGTAGCATTGAAGATATGGTTG-3′ Reverse: 3′-CAAGATGTCTCGGTTTACTAAGGGA-5′
IRF7	Forward: 5′-CTTCGTGATGCTGCGGGATAAC-3′ Reverse: 3′-CTGGTCTGACTCCGTCTCCG-5′
RSAD2	Forward: 5′-GAGGAGGTGGTGTAGGGATTATAGAG-3′ Reverse: 3′-GTTTCGTGATTTGGGACAGGCG-5′

### Statistical analysis

Averaged data were presented as mean ± SEM. Student’s *t*-test or Fisher’s exact test was used to examine the statistical significance. The significance level was set at *p* = 0.05.

## Results

### Participant demographics

Demographic and disease information were compared between PHN and HZ patients (**Table 1**). There was no significant difference in gender, age, pain scale, location, or chronic diseases (VAS). The trunk (thoracic nerve region) is the major skin location in both groups. Hypertension is the most frequent chronic disease in both groups. Regular blood examination found that there was a decreasing trend in white blood cells (*p* = 0.054) and basophils (*p* = 0.087) count, and an increasing trend in platelet large cell ratio (*p* = 0.067) (**Supplementary Table S1**). In addition, the neutrophil-to-lymphocyte ratio (NLR) was insignificantly decreased (from 4.73 to 3.38, *p* = 0.469). For HZ patients, nine out of 15 were cured and reported no pain 3 months later. Three of them reported a low level of pain (VAS < 3) while the other three reported a medium level of pain (3 < VAS < 6) 3 months later. For all eight PHN patients, a positive treatment had been received before the development of chronic pain.

### Overall transcriptomic comparison

**Figure 1:**
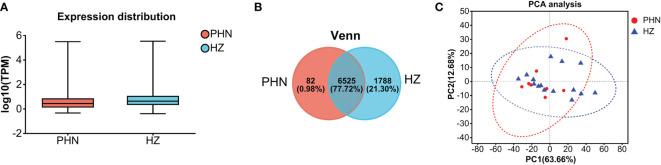
Comparison of overall transcriptomic profiling between PHN and HZ patients. **(A)** Distribution of transcriptomes detected; **(B)** Venn plot; **(C)** PCA analysis.

The expression of viral transcripts (ORF61, ORF63, and ORF66) was not detected in all the samples. As shown in **Figure 1A**, the distribution range of transcriptomic expression was similar between PHN and HZ patients. However, it seems the average level of expression was slightly lower in PHN compared to HZ. Moreover, there was less than 1% PHN-specific expression compared to more than 20% HZ-specific expression in the Venn plot (**Figure 1B**). On the other hand, PCA analysis did not separate the transcriptome of PHN from HZ. All the transcripts detected were included in **Supplementary File 1**.

### PHN-specific expression

**Figure 2:**
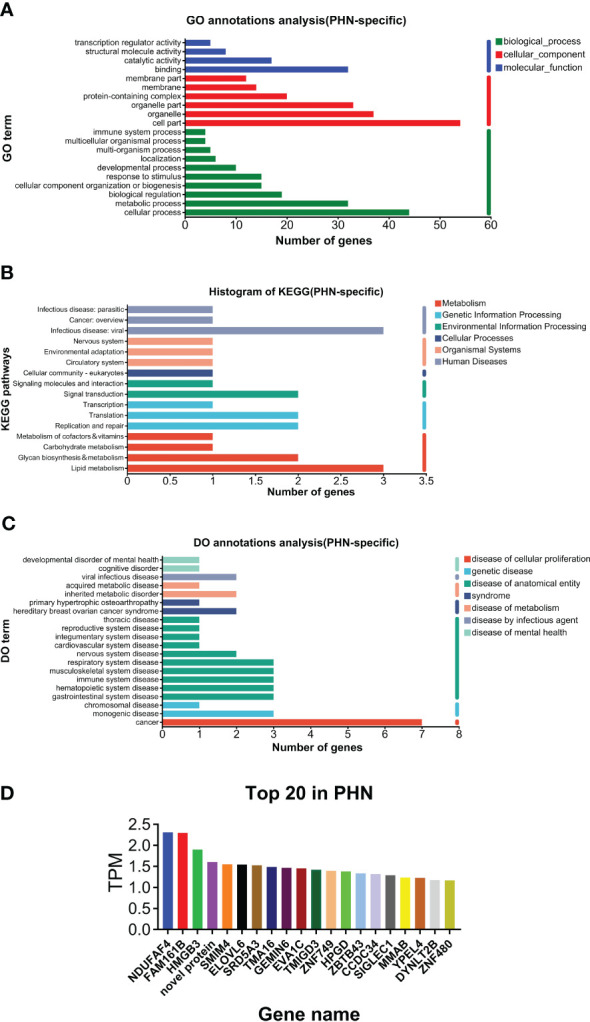
Analysis of PHN-specific expression. **(A)** GO analysis. **(B)** KEGG analysis. **(C)** DO analysis. **(D)** Top 20 expressed genes.

As shown in **Figure 2A**, GO analysis found that binding and catalytic activity, cell part and organelle, and cellular process and metabolic process were the top annotations for biological process, cellular components, and molecular function, respectively. KEGG analysis found that PHN-specific expression was more associated with infectious disease (viral), signal transduction, translation, replicate and repair, lipid metabolism, glycan biosynthesis and metabolism (**Figure 2B**). DO analysis found that cancer was the top disease expressing PHN-specific genes (**Figure 2C**). Moreover, functional analysis of top 20 genes revealed that a majority of them is involved in biosynthesis and metabolism (TMA16, HPGD, GEMIN6, SRD5A3, MMAB), immunity and inflammation (HMGB3, ELOVL6, TMIGD3, Siglec-1), and mitochondria respiratory chain (NDUFAF4, SMIM4) (**Figure 2D**). PHN-specific expression was included in **Supplementary File 1**.

### DEGs

**Figure 3:**
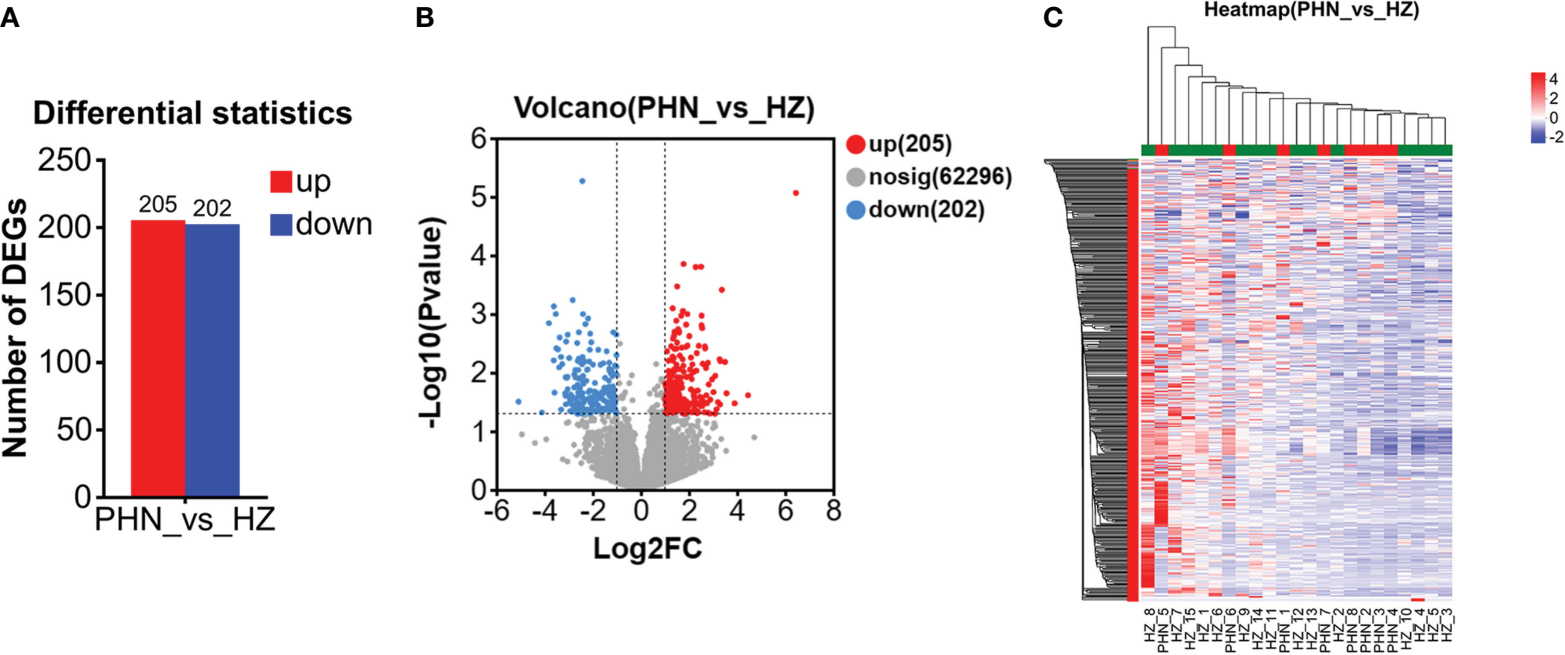
Comparison of overall DEG profiling between PHN and HZ patients. **(A)** Number of UP and DOWN DEG. **(B)**Volcano plot. **(C)** Heat map.

Among the 6,525 gene expressions detected in both PHN and HZ patients, there were 407 DEGs, including 205 upregulated DEGs (UP DEGs) and 202 downregulated DEGs (DOWN DEGs) (**Figure 3A**). The volcano distribution of UP and DOWN DEGs is shown in **Figure 3B**. Heatmap analysis found that half of the PHN patients were clustered on the right side, about one-sixth to one-third from the right end (**Figure 3C**). The other half spread out leftward with increasing distance (**Figure 3C**). All DEGs are listed in the **Supplementary File 1**.

**Figure 4:**
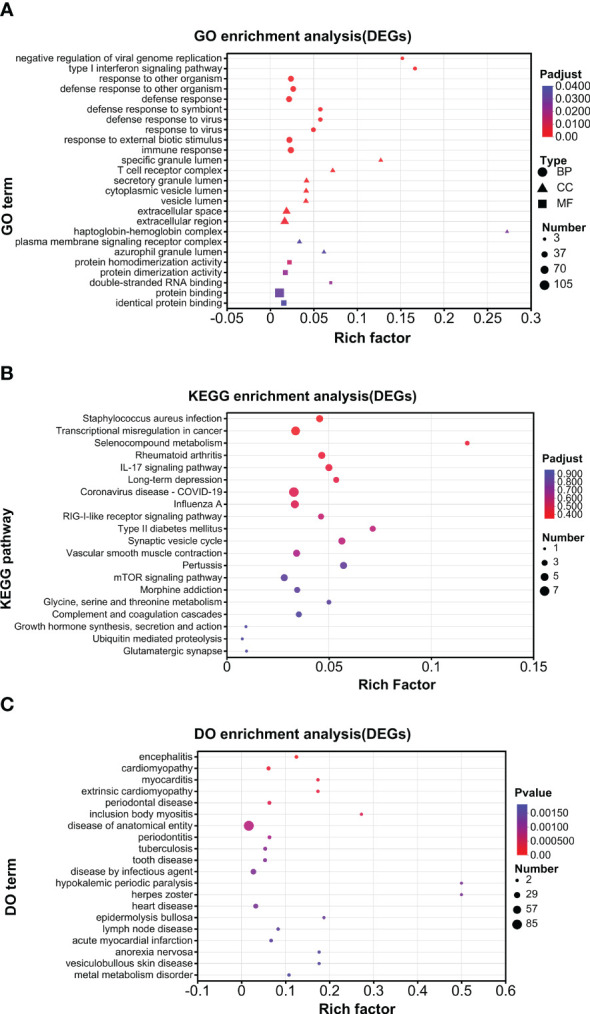
Enrichment analysis of DEGs. **(A)** GO analysis. **(B)** KEGG analysis. **(C)** DO analysis.

An enrichment analysis was conducted for DEGs. For all the DEGs (UP and DOWN DEGs), GO analysis found that negative regulation of viral genome replication and type I interferon signaling pathway were the top enriched biological processes; that specific granule lumen and haptoglobin–hemoglobin complex were the top enriched cellular components; and that 2′–5′-oligoadenylate synthetase activity and oxygen carrier activity were the top enriched molecular function (**Figure 4A**). KEGG analysis found that pertussis, synaptic vesicle cycle, type II diabetes mellitus, IL-17 signaling pathway, selenocompound metabolism, and long-term depression were enriched at higher levels (**Figure 4B**). DO analysis found that herpes zoster and hypokalemic periodic paralysis were the top diseases (**Figure 4C**).

Further enrichment analysis was conducted for UP DEGs and DOWN DEGs separately. For GO analysis, a similar pattern of annotations was observed between UP and DOWN DEGs (**Supplementary Figures S1A**, **2A**). For example, in both groups, cellular process and biological regulation, cell part and organelle, and binding and catalytic activity were the top 2 annotations for biological process, cellular component, and molecular function, respectively. For KEGG analysis, the overall pattern of annotations was similar between UP and DOWN DEGs (**Supplementary Figures S1B**, **2B**). For example, in both groups, infectious disease (viral.) and cancer (overview), immune system, cellular community (eukaryotes), signal transduction were the top annotations for human diseases, organismal system, cellular process, and environment information processing, respectively. However, endocrine and metabolic diseases and neurodegenerative diseases were selectively marked in the UP but not DOWN DEG groups and were among the top half annotations in the UP DEG group (**Supplementary Figure S1B**). Moreover, nucleotide metabolism was selectively in the UP DEG group, while metabolism of other amino acids and the metabolism of cofactors and vitamins were only in the DOWN DEG group (**Supplementary Figures S1B**, **S2B**). For DO analysis, the top 8 diseases were the same between UP and DOWN DEGs (**Supplementary Figures S1C**, **S2C**). However, nervous system disease ranked 2 and 8 in the UP and DOWN DEG groups, respectively (**Supplementary Figures S1C**, **S2C**).

**Figure 5:**
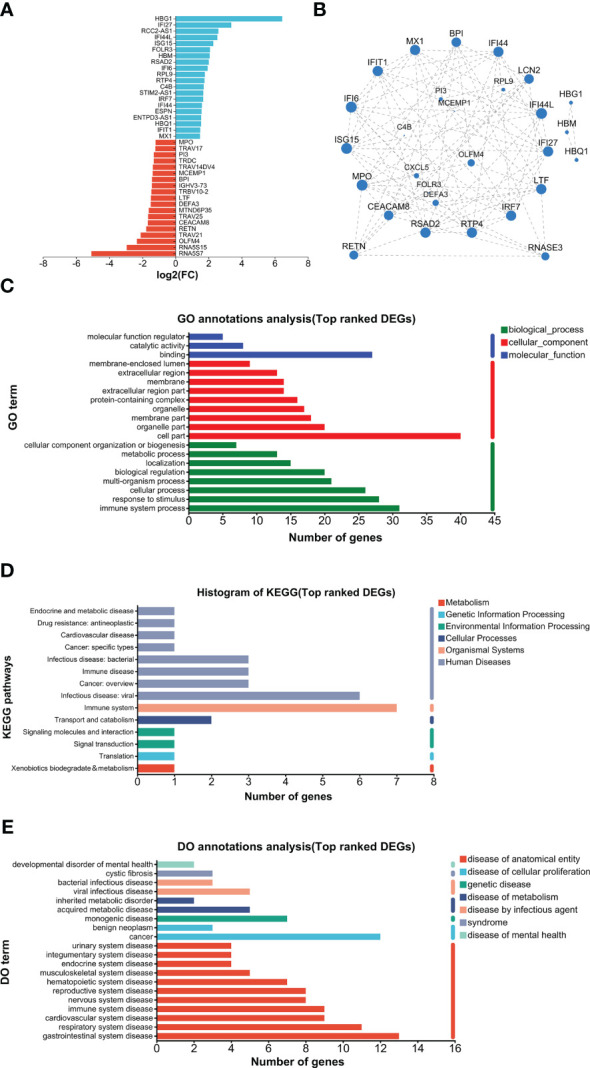
Analysis of top-ranked DEGs. **(A)** List of top-ranked DEGs. **(B)** PPI analysis. **(C)** GO analysis. **(D)** KEGG analysis. **(E)** DO analysis.

The top 20 UP and DOWN DEGs are listed in **Figure 5A**. The UP DEGs were increased by less than four to more than 100 times. The DOWN DEGs were reduced by about three- to30-fold (**Figure 5A**). GO analysis found that binding and cell part were the top annotations for molecular function and cellular component, respectively (**Figure 5C**). In addition, immune system processes, responses to stimulus, and cellular processes were the top 3 biological process (**Figure 5C**). For KEGG, immune system and infectious disease (viral) were the top 2 annotations, followed by infectious disease (bacteria), immune disease, and cancer (overview) (**Figure 5D**). DO analysis found that viral infection, acquired metabolic, monogenic, cancer, and multiple anatomical entity diseases were higher enriched (**Figure 5E**). PPI analysis found that major hub proteins included RTP4, IFI27, IFI44L, IFI44, LCN2, ISG15, MX1, IFIT1, LTF, RSAD2, RETN, MPO, CEACAM8, IFI6, IRF7, BPI, and RNASE3 (**Figure 5B**). UP and DOWN DEGs are listed in **Supplementary File 1**.

### HZ-specific expression

**Figure 6:**
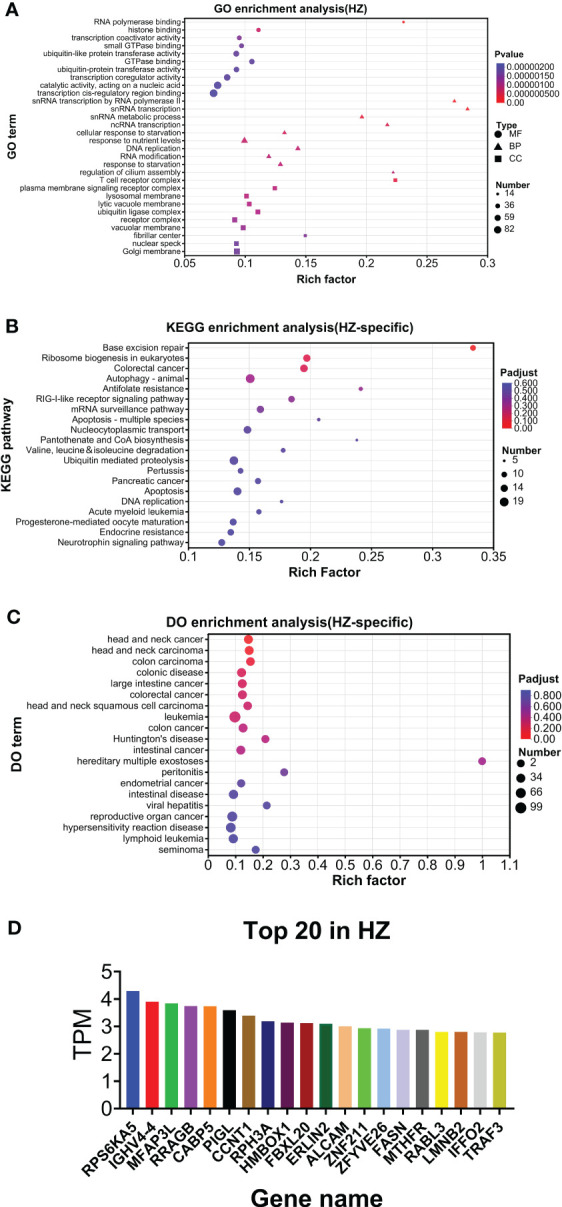
Analysis of HZ-specific expression. **(A)** GO analysis. **(B)** KEGG analysis. **(C)** DO analysis. **(D)** Top 20 expressed genes.

As shown in **Figure 6A**, GO analysis found that RNA polymerase binding and T-cell receptor complex were the top annotations for molecular function and cellular components, respectively. For biological processes, snRNA transcription by RNA polymerase II, snRNA transcription, snRNA metabolic process, ncRNA transcription, and regulation of cilium assembly were the top annotations (**Figure 6A**). KEGG analysis found that base excision repair, antifolate resistance, pantothenate and CoA biosynthesis, and apoptosis (multiple species) were highly enriched (**Figure 6B**). DO analysis found that hereditary multiple exostoses were the top enriched disease (**Figure 6C**). The top 20 genes are listed in **Figure 6D**. HZ-specific expression is listed in **Supplementary File 1**.

### CIBERSORT

**Figure 7:**
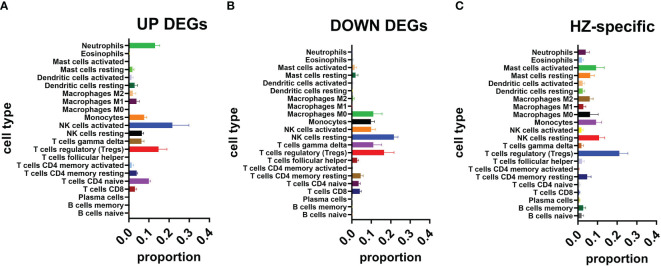
CIBERSORT analysis. **(A)** UP DEGS. **(B)** DOWN DEGs. **(C)** HZ-specific expression.

CIBERSORT analysis was conducted for DEGs and HZ-specific expression (**Figure 7**). Due to the small number of expressed genes, CIBERSORT was not conducted for PHN-specific expression.

CIBERSORT analysis for the UP DEG group was compared to the DOWN DEG group (**Figures 7A, B**). Among the 7 top-ranked cell types of the UP DEG group, neutrophils and naive CD4^+^ T cells were selective for the UP but not the DOWN group. The other 5 types of cells were in both UP and DOWN DEG groups including Treg, activated NK cell, monocytes, resting NK cell, and T cells gamma delta (Tγδ). In addition, M0 macrophages and resting memory CD4^+^ T cells were selectively ranked in the top 7 cell types of the DOWN but not the UP DEG group.

CIBERSORT analysis for HZ-specific expression found that the top 7-ranked cell types were T_reg_, NK resting cells, activated mast cells, monocytes, resting mast cells, M0 macrophages, and M2 macrophages (**Figure 7C**). Compared to the top 7-ranked cells in UP and DOWN DEG groups, T_reg_, resting NK cells, and monocytes were listed in all three groups, M0 macrophages were shared by HZ-specific expression and DOWN DEG groups; activated mast cells, resting mast cells and M2 macrophages were selectively seen in the HZ-specific expression group.

### qPCR validation

**Figure 8:**
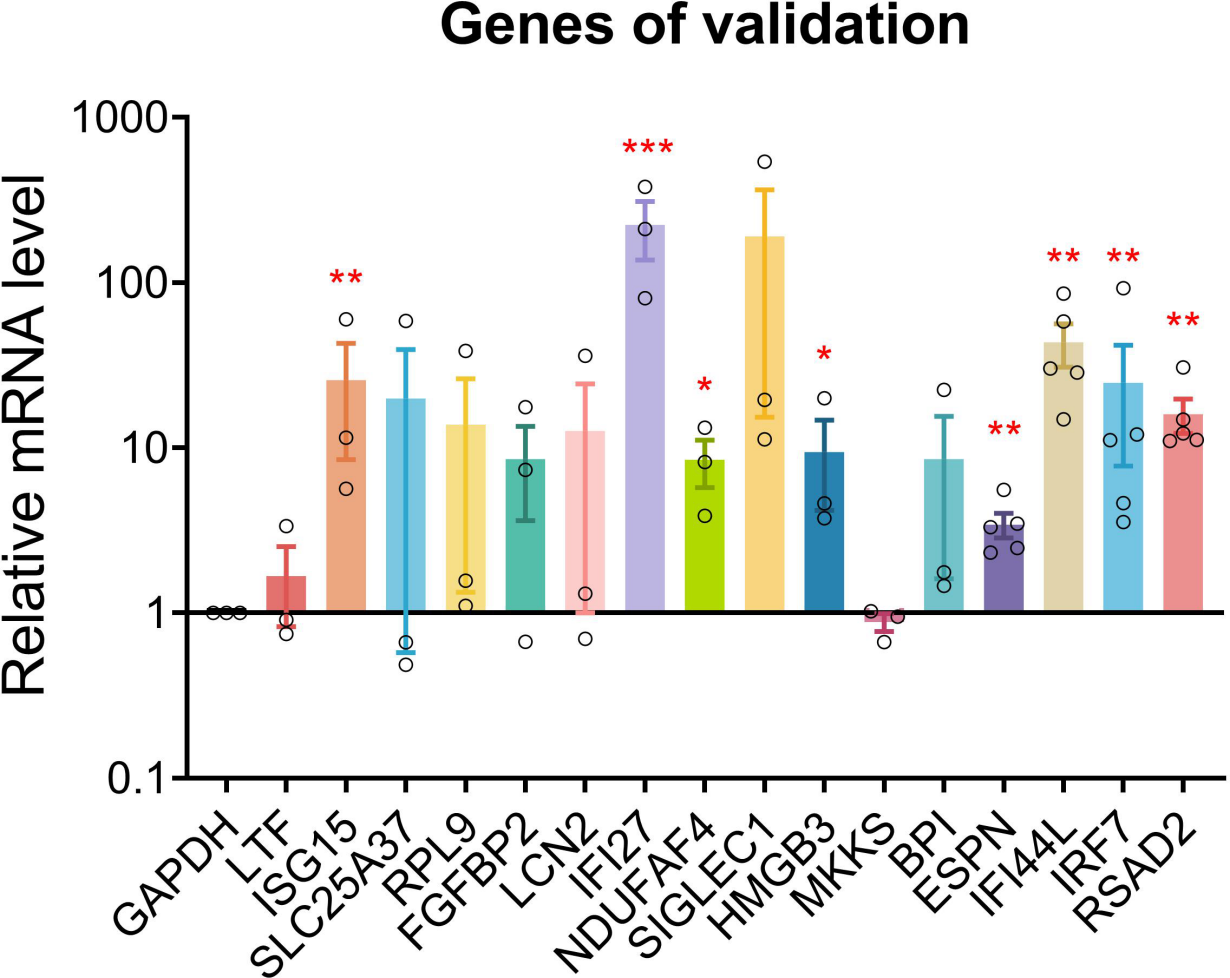
qPCR validation. qPCR validation was conducted for 17 genes between PHN and HZ groups. Data were expressed as mean ± SEM. ^*^ *p* < 0.05; ^**^ *p* < 0.01; ^***^, *p* < 0.001; Student’s *t*-test, PHN vs. HZ (*n* = 3–5).

qPCR was conducted for 16 genes. Most of the PHN-specific genes and UP DEGs were significantly or insignificantly increased in PHN compared to HZ groups (**Figure 8**). On the other hand, DOWN DEGs and HZ-specific genes were insignificantly decreased or increased in PHN compared to HZ patients (**Figure 8**).

### Comparison between five PHN and HZ patients

The expression profiles of five PHN patients (located in the lower left corner of PCA in **Figure 1**) and 15 HZ patients were compared preliminarily. As shown in **Supplementary Figure S3A**, the distribution range of transcriptomic expression was similar between PHN and HZ patients. However, the average level of expression was slightly lower in PHN compared to HZ. Moreover, there were only 13 PHN-specific expressions compared to 3,276 HZ-specific expressions in the Venn plot (**Supplementary Figure S3B**). PCA analysis is shown in **Supplementary Figure S3C**. PHN-specific and HZ-specific transcripts were included in **Supplementary File 2**.

Among the 5,037 gene expressions detected in both PHN and HZ patients, there were 487 DEGs, including 224 upregulated DEGs (UP DEGs) and 263 downregulated DEGs (DOWN DEGs) (**Supplementary Figure S4A**). The volcano distribution of UP and DOWN DEGs is shown in **Supplementary Figure S4B**. GO, KEGG, and DO analysis is shown in **Supplementary Figures S4C–E**. All DEGs were listed in Supplementary File 2. The top 20 UP and DOWN DEGs are listed in **Supplementary Figure S5A**. PPI analysis is shown in **Supplementary Figure S5B**. GO, KEGG, and DO analyses of the top 20 UP and DOWN DEGs are shown in **Supplementary Figures S5C–E**. All DEGs are included in **Supplementary File 2**.

Due to the limited number of PHN-specific genes. Enrichment analysis of these genes was not conducted. For the HZ-specific genes, GO, KEGG, and DO analyses were conducted (**Supplementary Figures S6A–C**). The top 20 genes of HZ-specific group are shown in **Supplementary Figure S6D**. CIBERSORT analysis for UP and DOWN DEGs and for the HZ-specific genes is shown in **Supplementary Figure S7**.

## Discussion

The overall transcriptomic data suggests that there is a reduced expression level in PHN compared to HZ in general (**Figures 1A, B**). Considering that HZ is a virus-induced inflammatory skin disease while PHN is free of zoster for months, it is not surprising that HZ would result in an upregulation of transcription related to systemic immunity and inflammation. In contrast, there are only 287 transcripts, including 82 PHN-specific expressions and 205 UP DEGs, compared to the 1,990 transcripts, which included 1,788 HZ-specific expressions and 202 DOWN DEGs (**Figures 1B**, **3A, B**). As the 287 transcripts display an opposite way of change (increase in PHN) compared to the 1,990 transcripts (decrease in PHN), it would be more likely to associate these increased transcripts with the development of PHN from HZ. On the other hand, the decreased transcripts could largely result from the disappearance of rash and the resolution of acute inflammation. Moreover, the evolution of HZ to PHN could be due to the progression of the diseases or to certain genetic characteristics that predispose some individuals to PHN. Considering the HZ patients develop much less severe pain 3 months later compared to the PHN patients in the current study, our analysis of genes increased in PHN may reflect the influence of a combination of both disease progression and genetic disposition on the development of PHN from HZ.

The PCA analysis does not separate the two patient groups in the current study (**Figure 1C**). It suggests that the major components of transcriptomic are not different between the PHN and HZ groups. This mixed distribution pattern could be due to the fact that both PHN and HZ involve the same ganglion neurons, similar dermatomes, and relatively small skin areas. However, in the heatmap of DEGs, half of the PHN patients are located side by side (**Figure 3C**). Moreover, these four clustered PHN patients, plus another PHN patient who is nearest to them in the heatmap, are all located in the same quarter of the PCA plot (down and left quarter) (**Figures 1C**, **3C**). These results suggest that there is an intrinsic homogeneity in transcriptome among PHN patients that enables them to be partially separated from the transcription pattern of HZ patients in the heatmap. On the other hand, the other three PHN patients are located in the other three quarters of the PCA plot, respectively. It may be related to the disease and demographic characteristics of PHN patients (**Figure 1C**; **Table 1**).

Enrichment analysis data suggest that PHN-specific expression participates in the lipid and glycan metabolism processes (**Figures 2B, C**). In addition, nucleotide metabolism and metabolic diseases are associated with UP DEGs (**Supplementary Figure S1B**). These results suggest that increased expression of genes in lipid, glycan, and nucleotide metabolism might be associated with and/or contribute to the development of PHN from HZ. A recent plasma metabolomic study suggests that there is an increase in amino acid metabolites in PHN compared to HZ patients (Zhou et al., 2022). Moreover, lipid metabolites are increased in PHN men, while amino acid metabolites are more pronouncedly increased in PHN women compared to their respective HZ counterparts. Therefore, increased metabolism overall, or in some category, might be associated with and/or contribute to the development of PHN from HZ overall or in some subpopulations of patients.

Several top-ranked PHN-specific genes encode proinflammatory molecules, including HMGB3, ELOVL6, TMIGD3, and Siglec-1 (**Figure 2D**). For example, Siglec-1 is a member of sialic acid-binding lectins of the Ig superfamily (Siglec) expressed in a subset of macrophages. On the one hand, Siglec-1 is involved in the virus infection (Mikulak et al., 2017; Perez-Zsolt et al., 2022). On the other hand, Siglec-1 mediates crosstalk between macrophages and other immune cells such as neutrophils, dendritic cells, and Tregs (Crocker et al., 1995 (Wu et al., 2009; Perez-Zsolt et al., 2022). Particularly, the Siglec-1-mediated interaction between macrophage and Tregs promotes inflammation through suppressing Tregs (Wu et al., 2021). This mechanism is associated with autoimmune diseases of the nervous system such as experimental autoimmune uveitis, experimental autoimmune encephalomyelitis, and neuronal ceroid lipofuscinoses (Jiang et al., 2006; Wu et al., 2009; Groh et al., 2016). These results might suggest that increased activation of Siglec-1 is associated with or contributes to the development of PHN from HZ.

A majority of the TOP UP DEGs are related to type I IFN signaling, including IFI27, IFI44L, ISG15, RSAD2, IFI6, IRF7, IFI44, and IFIT1. It has been suggested that type I IFN induces headache and flu-like pain symptoms in multiple sclerosis patients (Goldschmidt and Hua, 2020). Moreover, type I IFN therapy increases somatic pain scores in a subpopulation of chronic hepatitis C virus (HCV) patients who also develop depression following IFN treatment (Lin et al., 2020). A number of animal studies have been conducted to investigate type I IFN signaling of DRG neurons in chronic pain conditions (Donnelly et al., 2021; Wang et al., 2021). The majority of these studies suggest that upregulation of type I IFN signaling results in the sensitization of DRG neurons and chronic pain (Fitzgibbon et al., 2019; Sun et al., 2022; Wong et al., 2023; Zhang et al., 2023). Particularly, a recent study suggests that type I IFN directly acts on nociceptors to induce pain sensitization (Barragan-Iglesias et al., 2020). Moreover, the chemotherapy drug vinorelbine may induce pain by sequential activation of STING, pIRF3, type I IFN, mitogen-activated protein kinase interacting kinase (MNK), and eIF4E phosphorylation in DRG neuron (Franco-Enzastiga et al., 2024). Overall, our results suggest that activation of type I IFN signaling might play an important role in the development of PHN from HZ, possibly through effects on VZV-reactivated DRG neurons.

Six of the top 20 DOWN DEGs belong to T-cell receptors, including TRAV21, TRAV25, TRBV10-2, TRAV14DV4, TRDC, and TRAV17 (**Figure 5A**). Moreover, the T-cell receptor complex is a top annotation in GO analysis of HZ-specific expression. Multiple studies have investigated T cells in HZ patients. An earlier study has found no change in CD8^+^ T cells and a decrease in CD4^+^ T cells in HZ compared to control patients (Xing et al., 2013). In contrast, a recent study has found a transient increase of CD4^+^ and CD8^+^ T cells and B cells in the HZ stage (Peng et al., 2022). In addition, there is a significant decrease in CD4^+^ T cells in PHN compared to non-PHN groups (Peng et al., 2022). Partially similar to and different from each of the above two studies, another recent study has reported an increase in CD8^+^ T cells and a decrease in CD4^+^ T cells in HZ compared to control patients (Chen et al., 2023). These results might suggest that CD8^+^ T cells may be activated in the HZ stage compared to healthy controls. On the other hand, CD4^+^ T cells could be decreased in the HZ (vs. healthy control) and PHN (vs. non-PHN) stages. Moreover, our results suggest that there is an overall decrease in T cell activation in PHN compared to the HZ stage.

In addition to T-cell receptors, five of the top 20 DOWN DEGs are related to neutrophils, including DEFA3, LTF, BPI, MPO, and LCN2 (**Figure 5A**). In contrast, CIBERSORT analysis suggests that neutrophils are selectively associated with UP but not DOWN DEGs (**Figures 7A–C**). These results might suggest that the expression profile of neutrophils is altered in PHN compared to HZ stages. A recent study suggests that transient activation of neutrophils during the acute inflammation stage protects against the development of chronic low back pain (Parisien et al., 2022). Considering neutrophil infiltration is a common mechanism of chronic pain (Yang et al., 2022; Zhao et al., 2023), our study might suggest that persisted but altered activation of neutrophils contributes to the development of PHN from HZ.

On top of neutrophils, M1 and M2 macrophages are selectively associated with UP, while M0 macrophages are selectively associated with DOWN DEGs (**Figures 7A, B**). These results appear to suggest a transition from resting to activated states of macrophages from HZ to PHN. However, all M0, M1, and M2 macrophages are associated with HZ-specific genes (**Figure 7C**). Therefore, it might be suggested that there is a disappearance of M0 macrophages from HZ to PHN. Interestingly, the ratio of M1/M2 is higher in UP DEGs compared to the PHN-specific group (**Figures 7A, C**). As M1 macrophages are proinflammatory and cause pain, while M2 macrophages are anti-inflammatory and cause pain in general, our results might suggest that a higher M1/M2 ratio of macrophages contributes to the development of PHN in HZ.

Ramsay Hunt syndrome (RHS) is an acute peripheral facial neuropathy that occurs as a complication of shingles. It has been reported that patients with RHS who have an elevated NLR have poor outcomes, including a higher degree of paralysis and a lower probability of complete recovery (Soh et al., 2019). NLR is an independent risk factor for herpes zoster infection in patients with rheumatic diseases (Mok et al., 2023). Preoperative NLR is significantly associated with HZ and PHN in living donor liver transplantation recipients (Sim et al., 2021). In patients with HZ and malignancy, a higher NLR is associated with fatal outcomes (Yamamoto and Aoyama, 2023). These results suggest a higher NLR is associated with a higher frequency and worse outcomes of HZ or HZ-associated diseases. Our results (insignificant decrease of NLR in PHN) do not suggest whether or not a higher NLR may be critical in the development of PHN from HZ.

In addition to neutrophils, CIBERSORT analysis suggests that UP DEGs are associated with higher activated NK cells and a higher ratio of activated/resting NK cells compared to DOWN DEGs or HZ-specific groups (**Figures 7A–C**). Increased NK cells have been reported in HZ patients compared to healthy controls (Xing et al., 2013; Peng et al., 2022). However, NK cells are not different between PHN and non-PHN patients (Peng et al., 2022). Our results suggest that activated NK cells are increased in PHN compared to HZ stages. It has been reported that NK cells are negatively associated with mechanical pain sensitivity in chronic peripheral neuropathic pain conditions (Lassen et al., 2021). NK cells may help restore normal sensory function and reduce pain by eliminating miswired nerve endings in peripheral sensory neurons (Kim et al., 2023). Therefore, our results might suggest that abnormal axon degeneration/regeneration of DRG neurons contributes to the development of PHN in HZ.

There are several limitations to the current study. First, due to the lack of healthy controls and non-PHN patients (who had HZ previously), the current study cannot address how HZ develops from a healthy control or what differentiates PHN from non-PHN. Second, given the lack of a valid animal model for PHN (VZV selectively infects human cells), no animal experiments on effects and mechanisms were conducted. Finally, the study is limited by the small sample size and the presence of multiple chronic complications in the patients.

By comparing the blood transcriptomic signature of PHN to that of HZ patients, the current study suggests metabolism of lipid, glycan, and nucleotides, type I IFN signaling, and altered activation of neutrophils are associated with and potentially contribute to the development of PHN from HZ.

## Data Availability

The sequence data presented in the study are deposited in the NCBI Sequence Read Archive database, accession number PRJNA1142765.
