# Supplementary figures and images for: Exploring blood transcriptomic signatures in patients with herpes zoster and postherpetic neuralgia
Source: Front Cell Infect Microbiol. 2024 Aug 15;14:1425393. doi: 10.3389/fcimb.2024.1425393 (PMC11358128; doi:10.3389/fcimb.2024.1425393)

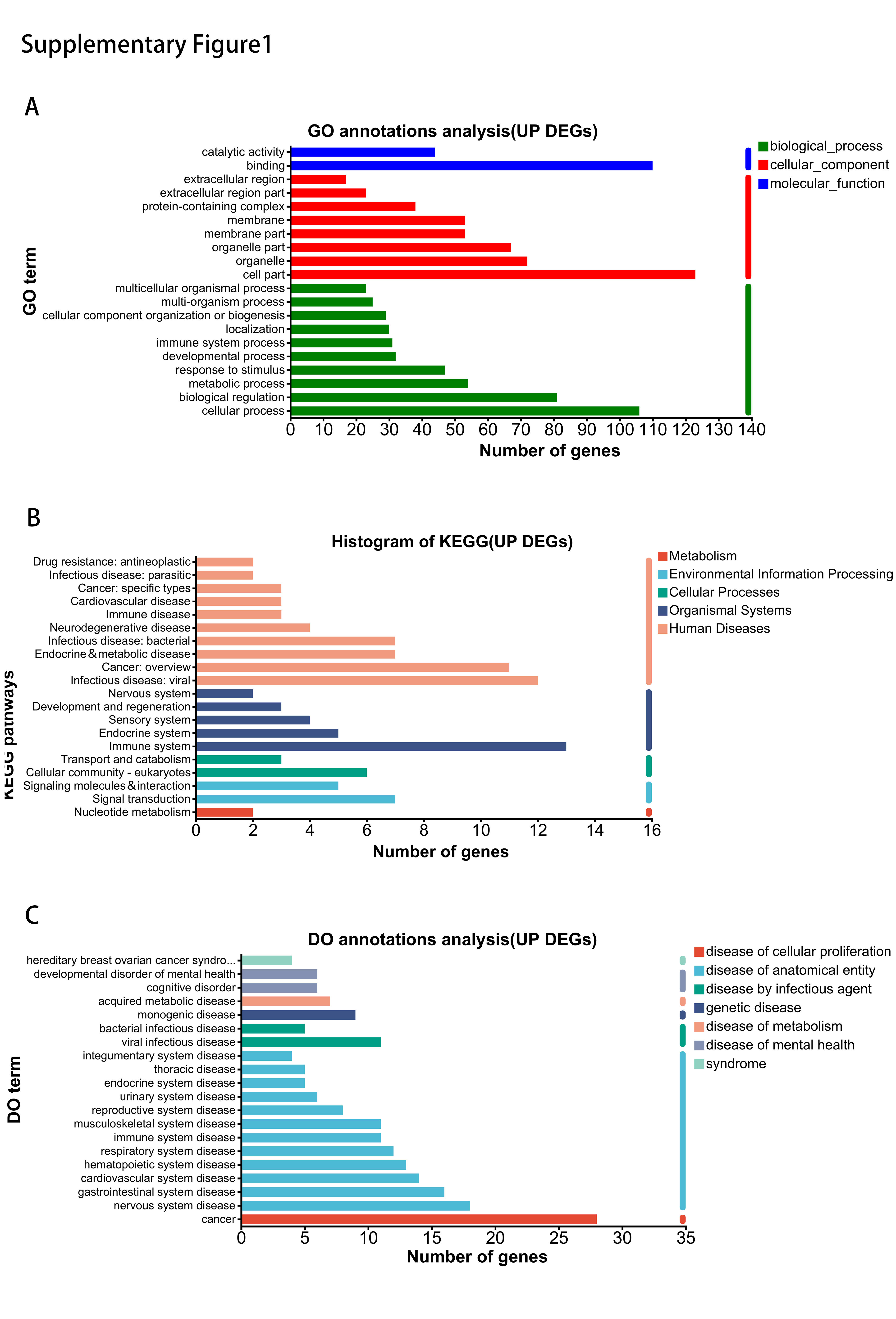

Supplement: Supplementary file 1 [file Image1.jpeg]

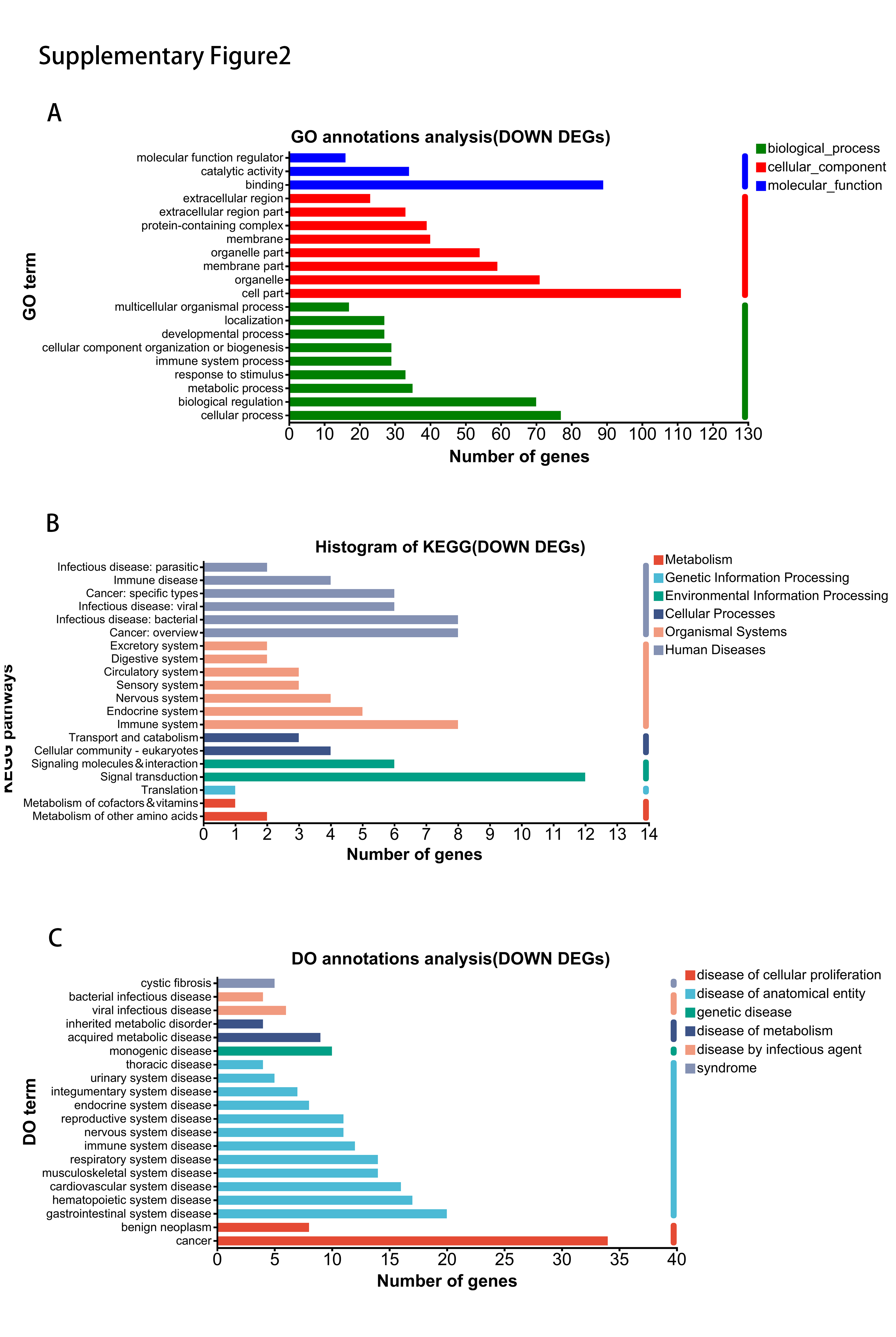

Supplement: Supplementary file 2 [file Image2.jpeg]

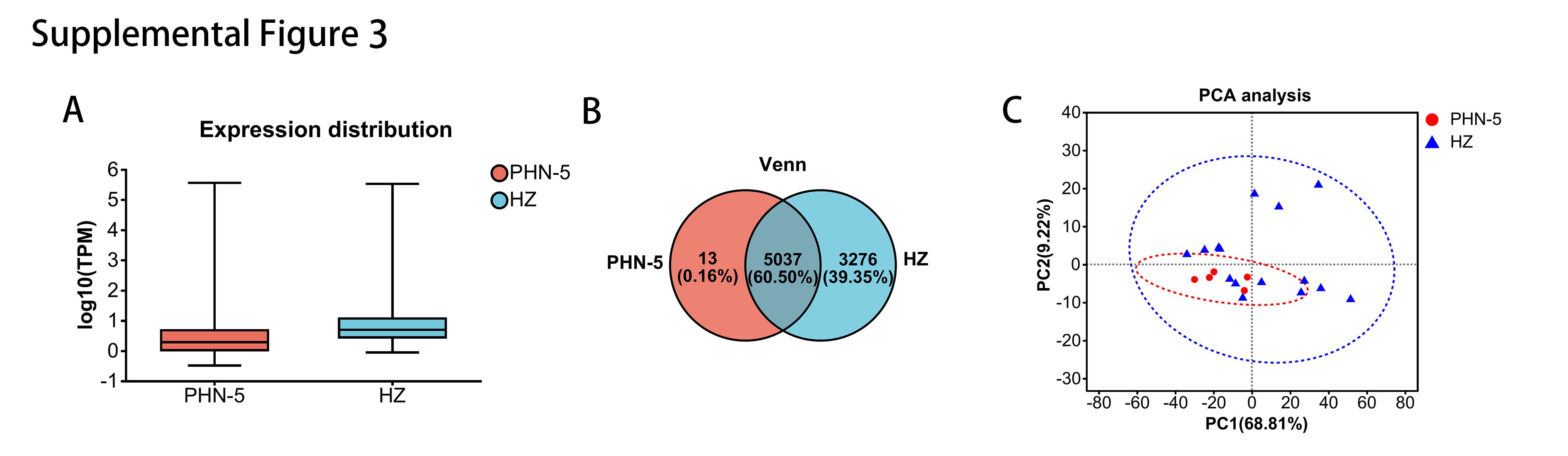

Supplement: Supplementary file 3 [file Image3.jpeg]

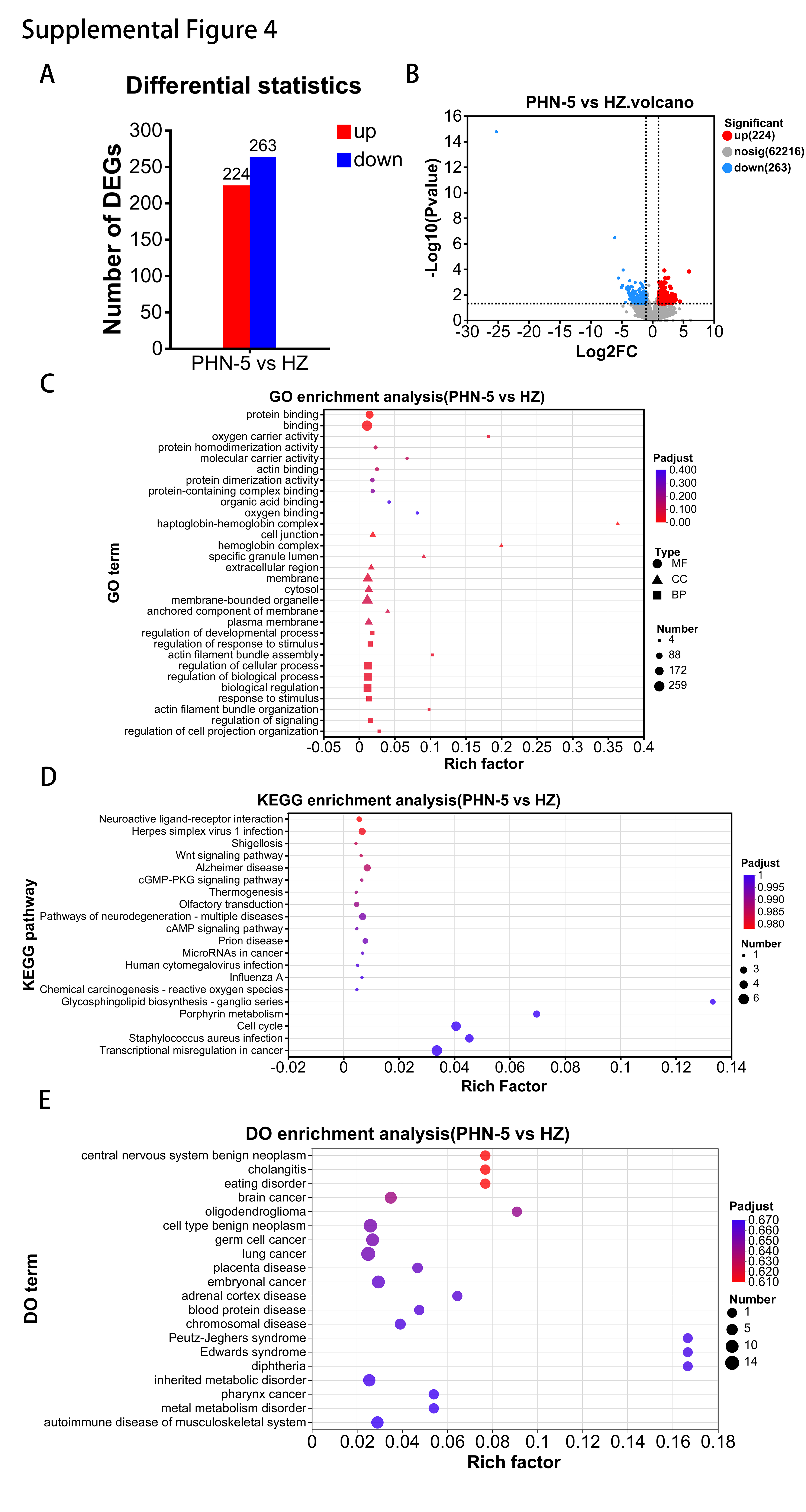

Supplement: Supplementary file 4 [file Image4.jpeg]

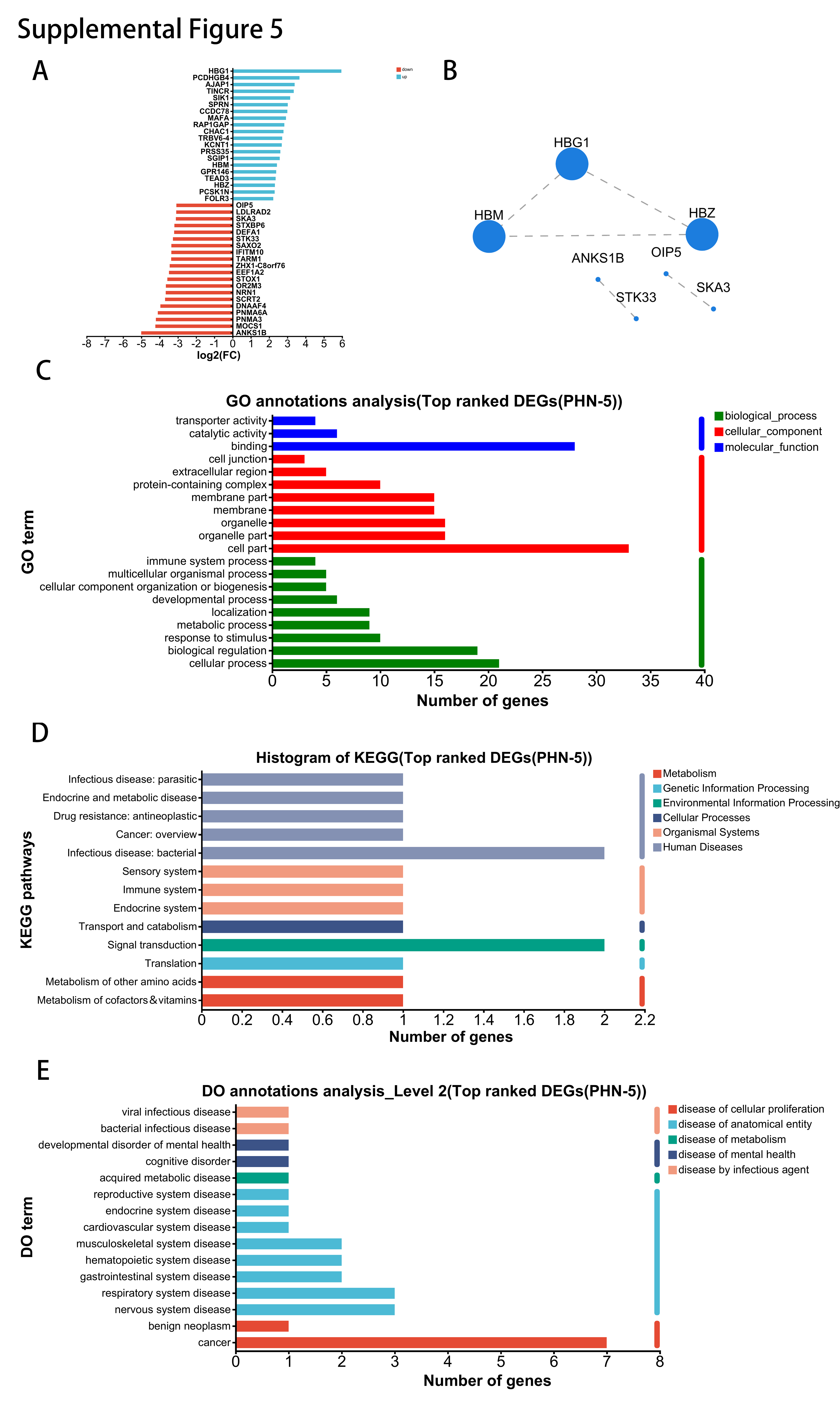

Supplement: Supplementary file 5 [file Image5.jpeg]

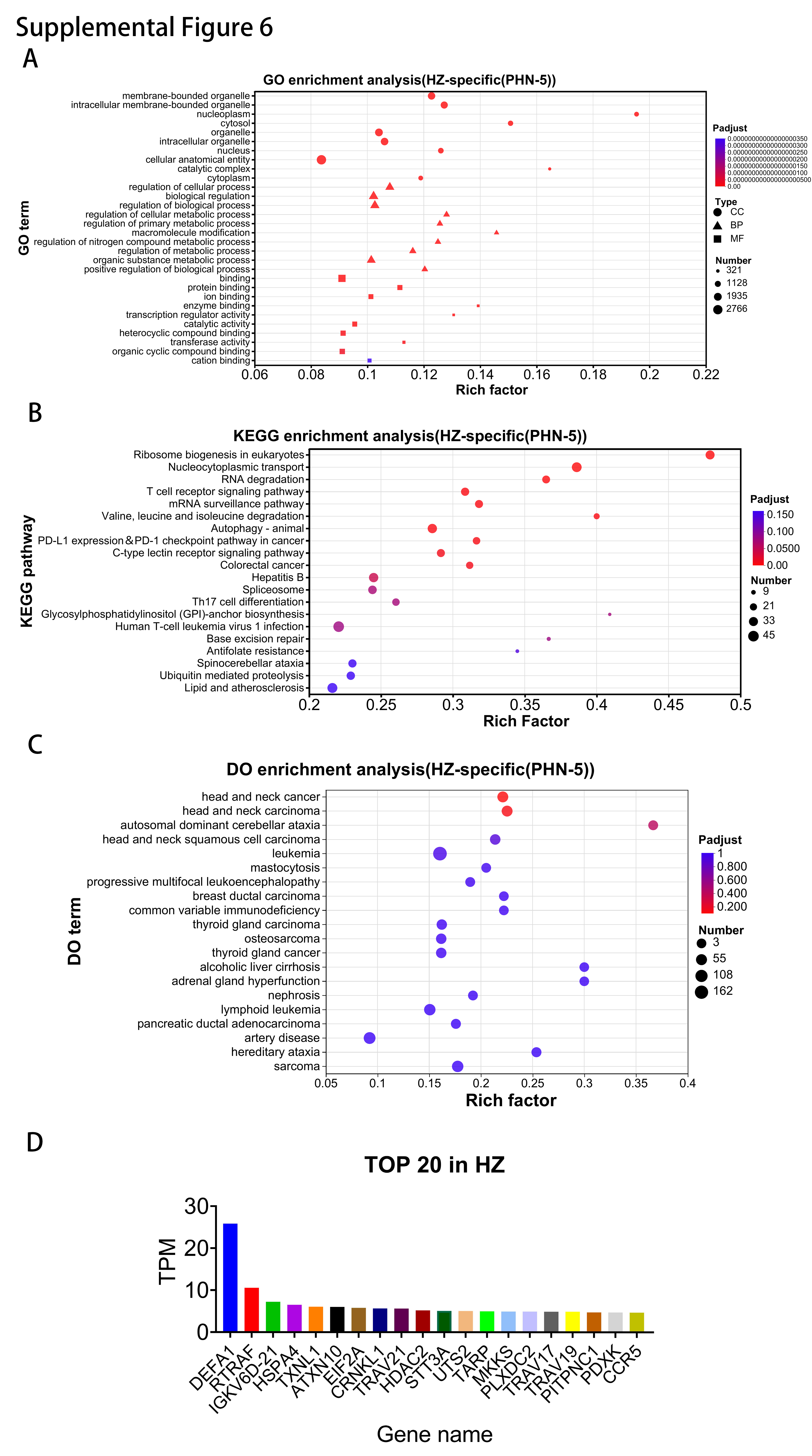

Supplement: Supplementary file 6 [file Image6.jpeg]

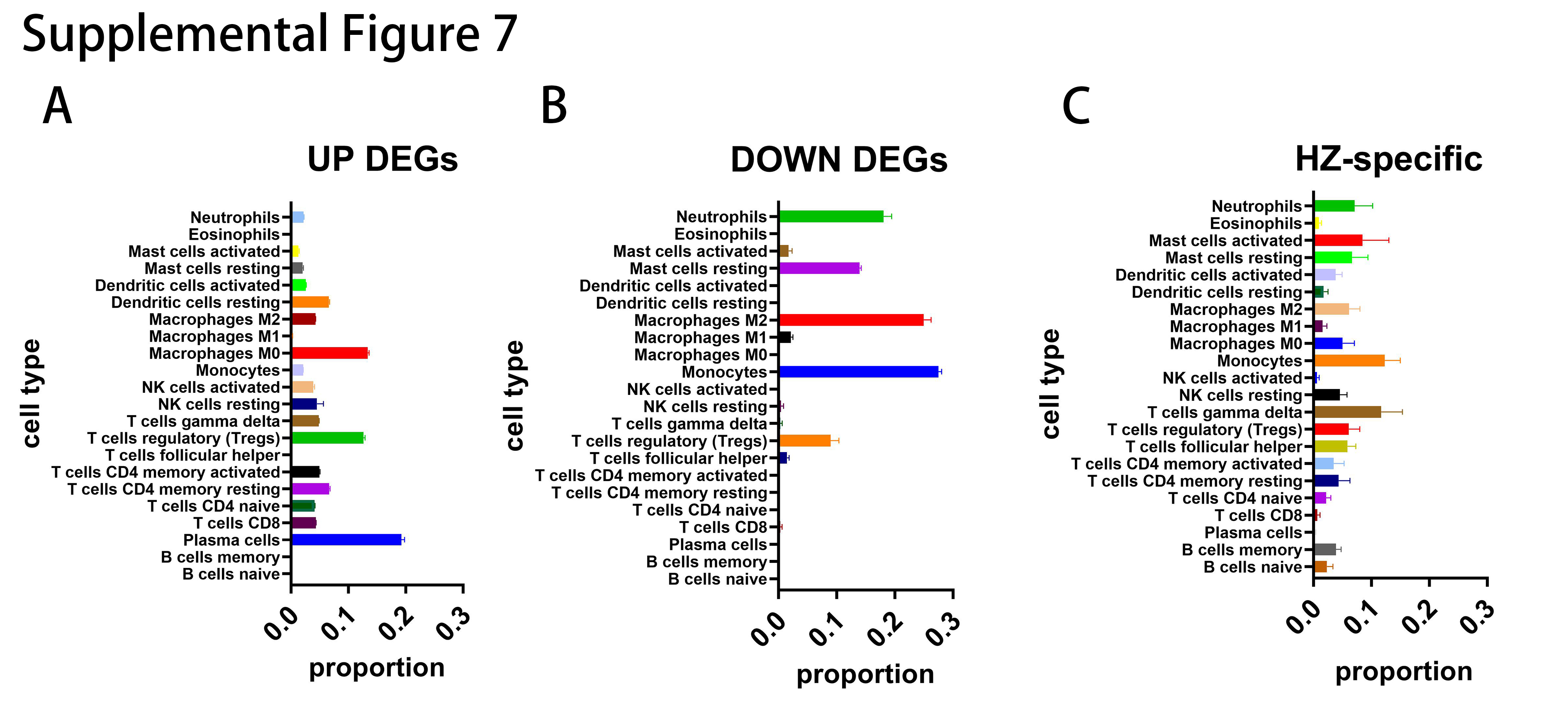

Supplement: Supplementary file 7 [file Image7.jpeg]
