# Supplementary material for: Exploring blood transcriptomic signatures in patients with herpes zoster and postherpetic neuralgia
Source: Front Cell Infect Microbiol. 2024 Aug 15;14:1425393. doi: 10.3389/fcimb.2024.1425393 (PMC11358128; doi:10.3389/fcimb.2024.1425393)
Supplement: Supplementary file 8 [file Table1.docx]

**Supplementary Table 1: Regular blood examination of PHN and HZ patients**

| group | PHN | | HZ | |  |
| --- | --- | --- | --- | --- | --- |
|  | MEAN | SE | MEAN | SE | P-value |
| White Blood Cell count | 6.49125×10^9^/L | 0.766877802 | 8.594×10^9^/L | 0.63221214 | 0.054561585 |
| Red Blood Cell count | 4.25125×10^12^/L | 0.221814252 | 4.14666666710^12^/L | 0.132232359 | 0.670284467 |
| Hemoglobin | 131.25g/L | 6.419139907 | 126g/L | 4.366539438 | 0.496691292 |
| Hematocrit | 39.05% | 1.597207384 | 38.04% | 1.189869941 | 0.619577049 |
| Erythrocyte mean corpuscular volume | 92.35fl | 1.834004206 | 89.66666667fl | 2.701122636 | 0.505707888 |
| Mean corpuscular hemoglobin | 30.9625pg | 0.529466544 | 30.40666667pg | 0.486921003 | 0.480094941 |
| Mean corpuscular hemoglobin concentration | 335.25g/L | 3.238992348 | 330.8g/L | 2.936145838 | 0.35231337 |
| Platelet count | 206.125×10^9^/L | 17.0487091 | 233.2666667×10^9^/L | 13.17192782 | 0.229587608 |
| %Lymphocyte | 27.9125% | 5.695406592 | 25.38% | 3.785476003 | 0.706595777 |
| %Monocyte | 7.9625% | 1.298342006 | 6.553333333% | 0.728786772 | 0.315711663 |
| %Neutrophil | 62.9875% | 6.506492293 | 66.52666667% | 4.274558921 | 0.643069852 |
| %Eosinophil | 0.775% | 0.23356706 | 1.146666667% | 0.231118742 | 0.314830844 |
| %Basophil | 0.3625% | 0.110093305 | 0.393333333% | 0.053866384 | 0.778911513 |
| NLR | 3.389883314 | 1.017523639 | 4.738161523 | 1.211818434 | 0.46908409 |
| Lymphocyte count | 1.84×10^9^/L | 0.559910707 | 1.994666667×10^9^/L | 0.298330061 | 0.790625516 |
| Monocyte count | 0.47×10^9^/L | 0.063723734 | 0.504×10^9^/L | 0.027581913 | 0.573417489 |
| Neutrophil count | 4.12125×10^9^/L | 0.710131873 | 5.970666667×10^9^/L | 0.773170016 | 0.133448461 |
| Eosinophil count | 0.0375×10^9^/L | 0.010133044 | 0.093333333×10^9^/L | 0.024585452 | 0.123553173 |
| Basophil count | 0.02×10^9^/L | 0.004225771 | 0.031333333×10^9^/L | 0.004007929 | 0.087249189 |
| RDW-CV | 13.55% | 0.46483484 | 13.29333333% | 0.348939436 | 0.66604713 |
| RDW-SD | 45.975fl | 1.973733771 | 44.34746667fl | 1.553830823 | 0.533809022 |
| Platelet volume distribution width | 11.975 | 0.76782392 | 10.66666667 | 0.401030419 | 0.108617678 |
| Mean platelet volume | 10.5fl | 0.322932987 | 9.250666667fl | 0.664383328 | 0.201896645 |
| Platelet hematocrit | 0.21875% | 0.022315714 | 0.237333333% | 0.009781745 | 0.384584927 |
| Platelet large cell ratio | 28.95% | 2.660491362 | 22.14666667% | 2.141447367 | 0.067034504 |
| %Nucleated red blood cell | 0.0125% | 0.0125 | 0.013333333% | 0.013333333 | 0.967972 |
| Nucleated red blood cell count | 0.00125×10^9^/L | 0.00125 | 0.000714286×10^9^/L | 0.000690066 | 0.691576739 |
